# Four new species in the *Tremellafibulifera* complex (Tremellales, Basidiomycota)

**DOI:** 10.3897/mycokeys.82.63241

**Published:** 2021-08-04

**Authors:** Long-Fei Fan, Renato Lúcio Mendes Alvarenga, Tatiana Baptista Gibertoni, Fang Wu, Yu-Cheng Dai

**Affiliations:** 1 Institute of Microbiology, School of Ecology and Nature Conservation, Beijing Forestry University, Beijing 100083, China; 2 College of Forestry, Beijing Forestry University, Beijing 100083, China; 3 Departamento de Micologia, Centro de Biociências, Universidade Federal de Pernambuco, Av. da Engenharia s/n, Recife, Pernambuco 50740-570, Brazil

**Keywords:** Multi-gene, phylogeny, taxonomy, Tremellaceae

## Abstract

Samples of species close to *Tremellafibulifera* from China and Brazil are studied, and *T.fibulifera* is confirmed as a species complex including nine species. Five known species (*T.cheejenii*, *T.fibulifera* s.s., *T.* “*neofibulifera*”, *T.lloydiae-candidae* and *T.olens*) and four new species (*T.australe*, *T.guangxiensis*, *T.latispora* and *T.subfibulifera*) in the complex are recognized based on morphological characteristics, molecular evidence, and geographic distribution. Sequences of eight species of the complex were included in the phylogenetic analyses because *T.olens* lacks molecular data. The phylogenetic analyses were performed by a combined sequence dataset of the internal transcribed spacer (ITS) and the partial nuclear large subunit rDNA (nLSU), and a combined sequence dataset of the ITS, partial nLSU, the small subunit mitochondrial rRNA gene (mtSSU), the translation elongation factor 1-α (TEF1), the largest and second largest subunits of RNA polymerase II (RPB1 and RPB2). The eight species formed eight independent lineages with robust support in phylogenies based on both datasets. Illustrated description of the six species including *Tremellafibulifera* s.s., *T.* “*neofibulifera*” and four new species, and discussions with their related species, are provided. A table of the comparison of the important characteristics of nine species in the *T.fibulifera* complex and a key to the whitish species in *Tremella* s.s. are provided.

## Introduction

*Tremella* Pers. is characterized by being parasitic on or associated with other fungi or lichens (Bandoni and Oberwinkler 1983; Chen 1998; Pippola and Kotiranta 2008; Malysheva et al. 2015; Zhao et al. 2019) and by having a haploid unicellular yeast stage and diploid stage in its life cycle (Boekhout et al. 2011; Weiss et al. 2014; Liu et al. 2015a; Zhao et al. 2019). *Tremella* s.l. includes approximately 90 species and about 50 are recognized as lichenicolous species (Kobayasi 1939; Bandoni 1958, 1987; Lowy 1971; Bandoni and Oberwinkler 1983; Diederich 1996; Chen 1998; Kirk et al. 2008; Pippola and Kotiranta 2008; Millanes et al. 2011, 2012, 2014, 2015, 2016; Malysheva et al. 2015; Zamora et al. 2016; Zhao et al. 2019). *Tremella* s.l. is polyphyletic and was divided into five groups by Chen (1998) including Mesenterica group, Fuciformis group, Indecorata group, Foliacea group and Aurantia group based on morphological characteristics and molecular data of ITS rDNA and nLSU rDNA sequencing. Then species in Mesenterica group and Fuciformis group were allocated to *Tremella* s.s., and species in Indecorata group, Foliacea group and Aurantia group were emended as *Pseudotremella* Xin Zhan Liu et al., *Phaeotremella* Rea and *Naematelia* Fr., respectively (Liu et al. 2015b; Wedin et al. 2016; Spirin et al. 2018). Besides, *Tremella* s.l. contained lichenicolous species that defined as *Tremella* clade I, clade II, clade III, and several single *Tremella* species lineages based on rDNA sequences (Millanes et al. 2011; Liu et al. 2015a, b).

Although *Tremella* s.l. was separated into several genera due to its polyphyletism, it is still somewhat confusing because taxonomic positions of some *Tremella* species are uncertain in Tremellales, especially some species recently described from lichens (Ariyawansa et al. 2015; Malysheva et al. 2015; Millanes et al. 2015; Zamora et al. 2016, 2018). These lichenicolous species were described as *Tremella*, but they were not clustered into *Tremella* s.s. in the phylogeny (Ariyawansa et al. 2015; Malysheva et al. 2015; Millanes et al. 2015; Zamora et al. 2016, 2018). Recently, Zhao et al. (2019) described four new *Tremella* species based on the phylogenetic relationship of 19 species in *Tremella* s.s., and Li et al. (2020) published a new yeast species of *Tremella* s.s. based on multi-gene analysis.

In this study, samples of species morphologically similar to *Tremellafibulifera* characterized by cerebriform whitish basidioma and abundant clamp complexes from China and Brazil are studied. Based on morphology, geographic distribution and phylogenetic analyses *T.fibulifera* is confirmed as a species complex, which was previously mentioned by Bandoni and Oberwinkler (1983) and Malysheva et al. (2015), and nine species are involved in the complex including five known species (*T.cheejenii*, *T.fibulifera* s.s., *T.* “*neofibulifera*”, *T.lloydiae-candidae* and *T.olens*) and four new species (*T.australe*, *T.guangxiensis*, *T.latispora* and *T.subfibulifera*) in the present study. The aim of this paper is to outline the *T.fibulifera* complex and describe two known species (*T.fibulifera* s.s., *T.* “*neofibulifera*”) and the four new species based on our collections.

## Materials and methods

### Sampling and morphological analysis

The studied specimens were collected from Rondônia and Pernambuco states in Brazil, Yunnan, Taiwan, Guangxi, Jilin Provinces in China. They are deposited at the herbaria of Beijing Forestry University (**BJFC**), Institute of Botânica in São Paulo (**SP**) and Universidade Federal de Pernambuco, Departamento de Micologia (**URM**). Macro-morphological illustrations refer to Chen (1998) and Zamora et al. (2017) and microscopic structures refer to Pippola and Kotiranta (2008) and Malysheva et al. (2015). Special color terms followed Petersen (1996). Handmade sections of dried basidioma were examined by a Nikon Eclipse 80i (Japan) microscope (magnification × 1000) after being mounted in 5% KOH for five minutes and treated with 1% Phloxine B (C_20_H_4_Br_4_Cl_2_K_2_O_5_). Microscopic structures were photographed using a Nikon Digital Sight DS-L3 (Japan) or Leica ICC50 HD (Japan) camera. Microscopic structures were examined and measured in the mix solution of 5% KOH and 1% Phloxine B. To represent variation in the size of spores, 5% of measurements were excluded from each end of the range, and are given in parentheses. Stalks were excluded for basidia measurements and apices were excluded for basidiospores measurements. Length and width of at least 30 basidia and basidiospores from each specimen were measured to micrometers.

Abbreviations as follows: **L** = mean length (arithmetic average of all basidia or spores length), **W** = mean width (arithmetic average of all basidia or spores width), **Q** = L/W ratio for each specimen studied, **n (a/b)** = number of spores (a) measured from given number of specimens (b).

### Molecular phylogeny

Dry specimens were used to extract DNA after pretreatment using TissuePrep (Jie Ling, China) by CTAB rapid plant genome extraction kit-DN14 (Aidlab Biotechnologies Co., Ltd, Beijing) or directly using the DNA easy Plant Mini Kit (Qiagen, China), according to the manufacturer’s instructions with some modifications. The internal transcribed spacer regions (**ITS**), partial nuclear large subunit rDNA (nLSU), the translation elongation factor 1-α (TEF1), the largest and second largest subunits of RNA polymerase II (RPB1 and RPB2), the small subunit mitochondrial rRNA gene (mtSSU) sequences were amplified with primer pairs listed in the Table 1. All newly generated sequences were submitted to GenBank (Table 2).

**Table 1.** Sequencing primers used in this study.

Pairs of primer	Nucleotide sequence (5′–3′)	Reference
**ITS**		
ITS5	5′–GGAAGTAAAAGTCGTAACAAGG–3′	White et al. 1990
ITS4	3′–TCCTCCGCTTATTGATATGC–5′	
ITS1	5′–TCCGTAGGTGAACCTGCGG–3′	
**Partial nLSU**		
LR0R	5′–ACCCGCTGAACTTAAGC–3′	Hopple and Vilgalys 1994
LR7	5′–TACTACCACCAAGATCT–3′	
**TEF1**		
983F	5′–GCYCCYGGHCAYCGTGAYTTYAT–3′	Rehner 2001
1567R	3′–ACHGTRCCRATACCACCRATCTT–5′	Rehner 2001
2218R	3′–ATGACACCRACRGCRACRGTYTG–5′	Rehner and Buckley 2005
**RPB1**		
Af	5′–GARTGYCCDGGDCAYTTYGG–3′	Stiller and Hall 1997
Cr	3′–CCNGCDATNTCRTTRTCCATRTA–5′	Matheny et al. 2002
**RPB2**		
5F	5′–GAYGAYMGWGATCAYTTYGG–3′	Matheny 2005
6F	5′–TGGGGKWTGGTYTGYCCTGC–3′	Matheny 2005
7R	3′–CCCATWGCYTGCTTMCCCAT–5′	Matheny 2005
7CR	3′–CCCATRGCTTGYTTRCCCAT–5′	Matheny 2005
Fcrypto	5′–TGGGGYATGGTTTGTCCKGC–3′	Ye et al. 2012
Rcrypto	3′–CCCATGGCTTGTTTRCCCATYGC–5′	Ye et al. 2012
**mtSSU**		
MS1	5′–CAGCAGTCAAGAATATTAGTCAATG–3′	White et al. 1990
MS2	3′–GCGGATTATCGAATTAAATAAC–5′	White et al. 1990

Polymerase chain reaction (**PCR**) cycling schedule for ITS, mtSSU and TEF1 included an initial denaturation at 95 °C for 3 min, followed by 35 cycles at 95 °C for 40 s, 54–56 °C (ITS) and 56–58 °C (mtSSU, TEF1) for 45 s, 72 °C for 1 min, and a ﬁnal extension at 72 °C for 10 min, for RPB1 and RPB2 included an initial denaturation at 95 °C for 3 min, followed by 9 cycles at 94 °C for 45 s or 1 min , 58 °C for 45 s or 60 °C for 1 min and 72 °C for 1.5 min, then followed by 35 cycles at 95 °C for 1 min, 53 °C or 55 °C for 45 s and 72 °C for 1 min, and a ﬁnal extension of 72 °C for 10 min, for partial nLSU included an initial denaturation at 94 °C for 1 min, followed by 34 cycles at 94 °C for 30 s, 50–52 °C for 1 min, 72 °C for 1.5 min, and a final extension at 72 °C for 10 min. PCR products were purified at the Beijing Genomics Institute (BGI), China or at the Plataforma Tecnológica de Genômica e Expressão Gênica do Centro de Biociências (UFPE) with the same primers.

Newly generated sequences in this study were aligned with additional related sequences downloaded from GenBank (Table 2) using MAFFT 7.0 online service with the Q-INS-i strategy (Katoh et al. 2019, http://mafft.cbrc.jp/alignment/server/). Prior to phylogenetic analysis, ambiguous sequences at the start and the end were deleted and gaps were manually adjusted to optimize the alignment using the default parameters in BioEdit (Hall 1999). Those positions deemed ambiguous to align were excluded manually. Multi-genes were concatenated as a combined file by Mesquite version 3.2. (Maddison and Maddison 2017). Phylogenetic analyses were applied to the ITS + partial nLSU dataset and the combined ITS+partial nLSU+mtSSU+TEF1+RPB1+RPB2 dataset. Sequences of *Cryptococcusdepauperatus* (Petch) Boekhoutet et al. were used as outgroup, which referred to Malysheva et al. (2015). The final concatenated sequence alignments were deposited in TreeBase (https://treebase.org/treebase-web/home.html; submission ID 28280 for ITS + partial nLSU dataset; submission ID 27553 for ITS + partial nLSU + mtSSU + TEF1 + RPB1 + RPB2 dataset) and the taxonomic novelties in MycoBank (http://www.MycoBank.org).

**Table 2.** Information on sequences used in this study.

Species	Sample no.	GenBank accessions						References
		ITS	Partial nLSU	mtSSU	TEF1	RPB1	RPB2	
***Tremellaaustral* sp. nov.**	**Dai 11539**	**MT445847**	–	–	**MT445759**	–	–	**Present study**
***T.austral* sp. nov.**	**Wu 154**	**MT445848**	**MT425188**	**MT483749**	**MT445760**	–	**MT445753**	**Present study**
* T. basidiomaticola *	CBS 8225	MH712822	MH712786	–	–	–	–	Zhao et al. 2019
* T. basidiomaticola *	CGMCC 2.5724^T^	MH712820	MH712784	–	–	–	–	Zhao et al. 2019
* T. basidiomaticola *	CGMCC 2.5725	MH712821	MH712785	–	–	–	–	Zhao et al. 2019
* T. brasiliensis *	CBS 6966^R^	AF444429	AF189864	KF036694	KF037200	–	KF036938	Liu et al. 2015a
* T. brasiliensis *	CBS 8212	KY105674	KY109886	–	–	–	–	Vu et al. 2016
* T. cerebriformis *	ZRL 20170101	MH712823	MH712787	–	–	–	–	Zhao et al. 2019
* T. cerebriformis *	ZRL 20170269	MH712824	MH712788	–	–	–	–	Zhao et al. 2019
* T. cheejenii *	GX 20172598	MH712825	MH712789	–	–	–	–	Zhao et al. 2019
* T. cheejenii *	GX 20172640	MH712826	MH712790	–	–	–	–	Zhao et al. 2019
* T. dysenterica *	LE 303447	KP986509	KP986542	–	–	–	–	Malysheva et al. 2015
* T. dysenterica *	VLA M 18599	KP986531	–	–	–	–	–	Malysheva et al. 2015
* T. erythrina *	HMAS 255317	MH712827	MH712791	–	–	–	–	Zhao et al. 2019
* T. erythrina *	HMAS 279591	MH712828	MH712792	–	–	–	–	Zhao et al. 2019
***T.fibulifera* s.s.**	**SP 211759**	**MT445850**	**MT425190**	**MT483750**	–	–	–	**Present study**
***T.fibulifera* s.s.**	**Alvarenga 471**	**MT445851**	**MT425191**	–	–	–	–	**Present study**
* T. flava *	CBS 8471^R^	KY105681	KY105681	KF036699	KF037205	KF036527	KF036943	Liu et al. 2015a
* T. flava *	CCJ 907	AF042403	AF042221	–	–	–	–	Zhao et al. 2019
* T. fuciformis *	CBS 6970^R^	KY105683	AF075476	KF036701	KF037207	KF036529	–	Liu et al. 2015a
* T. fuciformis *	CCJ1080	AF042410	AF042228	–	–	–	–	Malysheva et al. 2015
* T. globispora *	CBS 6972^R^	AF444432	AF189869	KF036703	KF037208	KF036531	KF036947	Liu et al. 2015a
* T. globispora *	UBC 586	AF042425	AF042243	–	–	–	–	Zhao et al. 2019
***T.guangxiensis* sp. nov.**	**Wu 3**	**MT445843**	**MT425186**	**MT483748**	**MT445756**	**MT445746**	**MT445752**	**Present study**
***T.guangxiensis* sp. nov.**	GX 20172028	MH712829	MH712793	–	–	–	–	Zhao et al. 2019
***T.latispora* sp. nov.**	**Dai 17574**	**MT445852**	**MT425192**	**MT483751**	**MT445761**	**MT445750**	**MT445754**	**Present study**
***T.latispora* sp. nov.**	**Dai 17568**	**MT445853**	**MT425193**	**MT483752**	**MT445762**	**MT445751**	**MT445755**	**Present study**
*T.laurisilva*e	S-F 102408(AM4)	JN053467	JN043572	–	–	–	–	Zhao et al. 2019
* T. lloydiae-candidae *	VLA M 11702	KP986536	KP986559	–	–	–	–	Malysheva et al. 2015
* T. lloydiae-candidae *	VLA M 11703	KP986559	KP986560	–	–	–	–	Malysheva et al. 2015
* T. mesenterica *	CBS 6973^R^	AF444433	AF444433	KF036705	KF037210	KF036533	KF036949	Liu et al. 2015a
* T. mesenterica *	FO 24610	AF042447	AF042265	–	–	–	–	Zhao et al. 2019
***T. “neofibulifera***”	**Wu 248**	**MT445844**	**MT425187**	–	**MT445757**	**MT445747**	–	**Present study**
***T. “neofibulifera***”	**Wu 243**	**MT445845**	–	–	–	**MT445748**	–	**Present study**
***T. “neofibulifera***”	**Wu 244**	**MT445846**	–	–	**MT445758**	**MT445749**	–	**Present study**
***T. “neofibulifera***”	LE 303445	KP986518	KP986547	–	–	–	–	Malysheva et al. 2015
* T. resupinata *	CBS 8488^T^	AF042421	AF042239	KF036708	KF037212	KF036535	KF036951	Liu et al. 2015a
* T. saccharicola *	DMKU-SP23^T^	AB915385	AB909021	–	–	–	–	Khunnamwong et al. 2019
* T. saccharicola *	DMKU-SP40	AB915386	AB909022	–	–	–	–	Khunnamwong et al. 2019
* T. salmonea *	GX 20172637	MH712851	MH712815	–	–	–	–	Zhao et al. 2019
* T. samoensis *	LE 303465	KP986508	KP986541	–	–	–	–	Malysheva et al. 2015
* T. samoensis *	VLA M 18603	KP986532	KP986555	–	–	–	–	Malysheva et al. 2015
* T. shuangheensis *	CBS 15561	MK050285	MK050285	MK050285	MK849087	MK849223	MK849362	Li et al. 2020
***T.subfibulifera* sp. nov.**	**Alvarenga 334**	**MT445849**	**MT425189**	–	–	–	–	**Present study**
* T. taiwanensis *	CBS 8479^R^	AF042412	AF042230	KF036709	KF037213	KF036536	KF036952	Liu et al. 2015a
* T. taiwanensis *	GX 20170625	MH712854	MH712818	–	–	–	–	Zhao et al. 2019
* T. tropica *	CBS 8483^R^	KY105697	KY109908	KF036710	KF037214	KF036537	KF036953	Liu et al. 2015a
* T. tropica *	CBS 8486	KY105696	KY109909	–	–	–	–	Liu et al. 2015a
* T. yokohamensis *	JCM 16989	HM222926	HM222927	–	–	–	–	Zhao et al. 2019
* T. yokohamensis *	CBS 11776	KY105698	KY109910	–	–	–	–	Malysheva et al. 2015
* Cryptococcus depauperatus *	CBS 7841^T^	FJ534881	FJ534911	AJ568017	KF037150	KF036471	–	Zhao et al. 2019

The samples used in this study are in bold.

Phylogenetic constructions of Maximum likelihood (ML), Maximum parsimony (MP), and Bayesian analyses were performed in the CIPRES Science Gateway portal Version 3.3 (Miller et al. 2012) using tool of RAxML-HPC BlackBox 8.2.6, PAUP on XSEDE (4.a165) and Mrbayes on XSEDE 3.2.6 respectively. All characters were equally weighted, and gaps were treated as missing data. Trees were inferred using heuristic search option with TBR branch swapping and 1000 random sequence additions. MrModeltest 2.3 (Posada and Crandall 1998; Nylander 2004) was used to determine the best-fit evolution model for both datasets for Bayesian analyses using MrBayes3.1.2 (Ronquist and Huelsenbeck 2003). Four Markov chains were run for two runs from random starting trees for 3 million generations (ITS + nLSU) and for 5 million generations (ITS + partial nLSU + mtSSU + TEF1 + RPB1 + RPB2) until the split deviation frequency value < 0.01, and trees were sampled every 100 generation. The first quarter generations were discarded as burn-in. A majority rule consensus tree of all remaining trees was calculated.

Phylogenetic trees were viewed by FigTree v. 1.4.2 (Rambaut 2012) and edited by Adobe Illustrator CS6 (Guide 2012). Branches that received bootstrap support for Maximum parsimony (BP), Maximum likelihood (BS) and Bayesian posterior probabilities (BPP) greater than or equal to 50% (BP/BS) and 0.95 (BPP) were considered as significantly supported, respectively.

## Results

### Phylogeny

**Figure 1.**
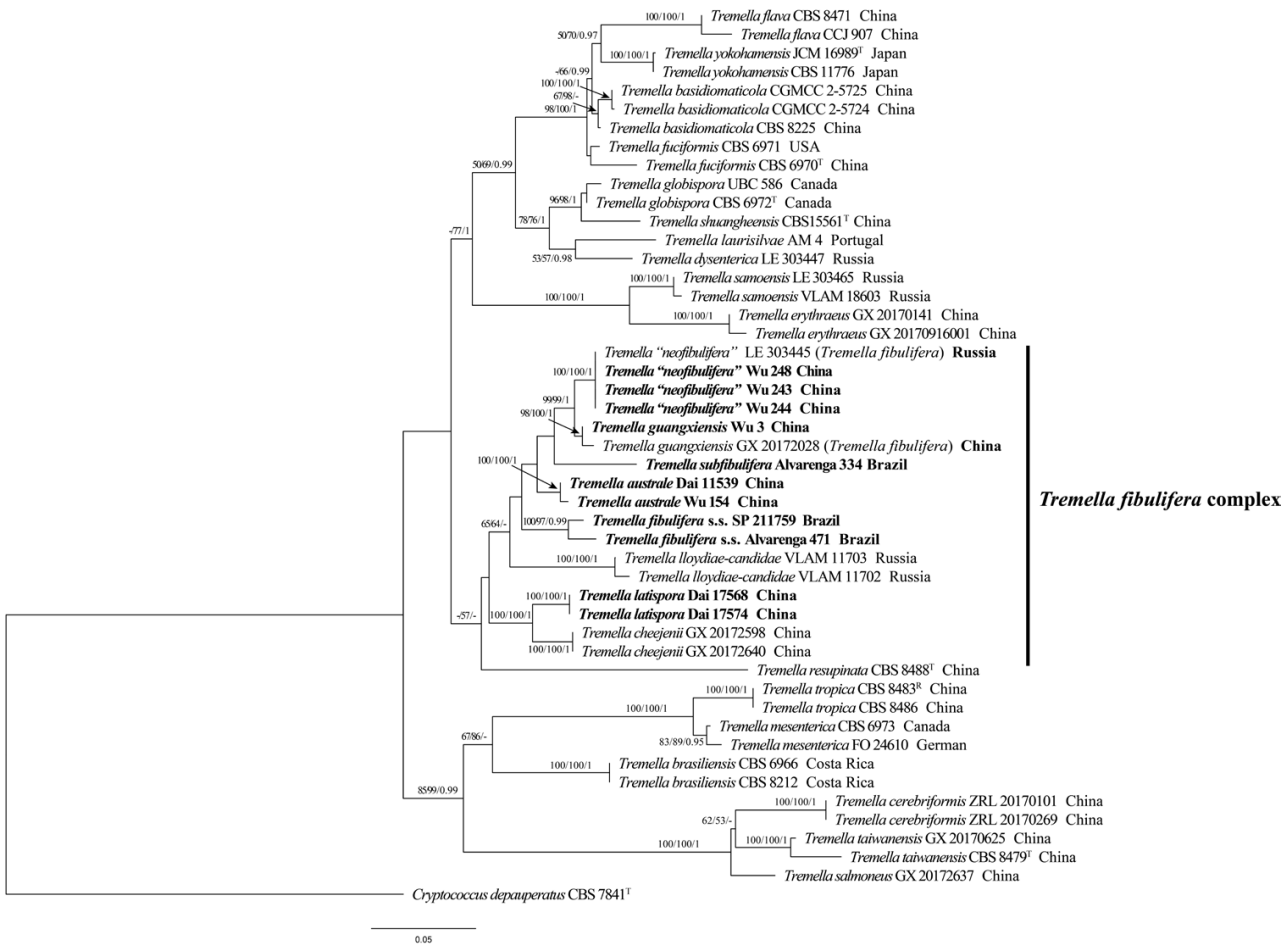
The Maximum likelihood tree showing phylogenetic relationship of species in *Tremella* s.s. based on the ITS + partial nLSU dataset. Bootstrap support values for MP and ML greater than 50% and BI greater than 0.95 are given at each node respectively. The samples used in this study are in bold.

The ITS + partial nLSU dataset included 50 fungal specimens representing 27 species. The dataset has an aligned length of 2282 total characters including gaps, of which 1777 characters are constant, 128 variable characters are parsimony-uninformative, and 377 are parsimony-informative. MP analysis yielded four equally parsimonious trees (TL = 1394, CI = 0.529, RI = 0.792, RC = 0.419, HI = 0.471). The best model for the ITS + partial nLSU dataset estimated and applied in the BI analysis was GTR. BI and ML analyses generated similar topologies as MP analysis, with an average standard deviation of split frequencies = 0.002648 (BI). The best tree obtained from the ML analysis with bootstrap values for BP, BS and BPP is shown in Fig. 1. The phylogeny shows that eight species are clustered into the *T.fibulifera* complex and four new species form four independent lineages with robust support.

**Figure 2.**
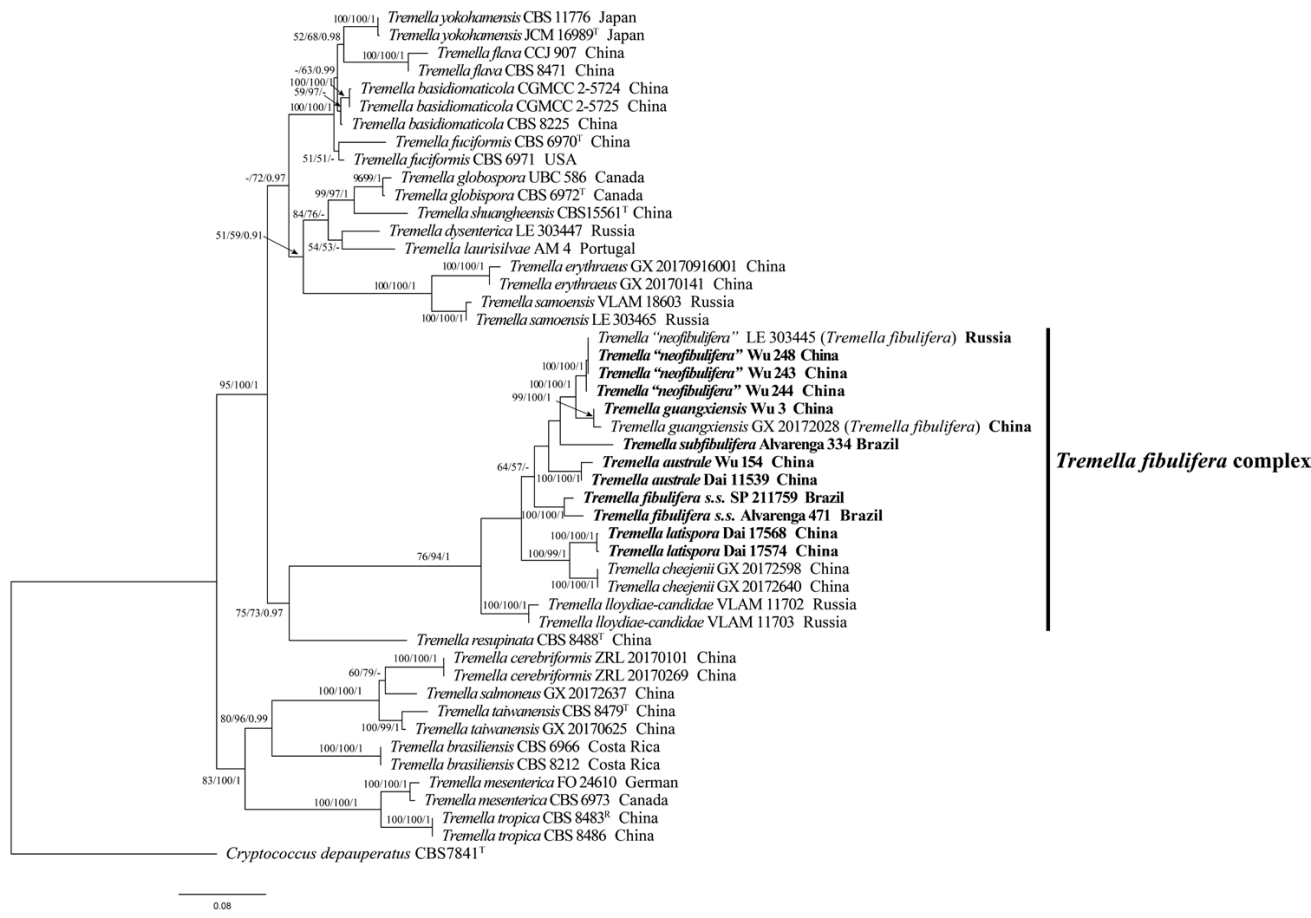
The Maximum likelihood tree showing phylogenetic relationship of species in *Tremella* s.s. based on the combined ITS + partial nLSU + mtSSU + TEF1 + RPB1 + RPB2 dataset. Bootstrap support values for MP and ML greater than 50% and BI greater than 0.95 are given at each node respectively. The samples used in this study are in bold.

The combined dataset of ITS + partial nLSU + mtSSU + TEF1 + RPB1 + RPB2 has an aligned length of 5113 total characters including gaps, of which 3332 characters are constant, 383 variable characters are parsimony-uninformative, and 1398 are parsimony-informative. MP analysis yielded two equally parsimonious trees (TL = 4519, CI = 0.607, RI = 0.730, RC = 0.443, HI = 0.393). The best model for the combined dataset estimated and applied in the BI analysis was GTR+I+G. BI analysis generated similar topology to MP and ML analysis, with an average standard deviation of split frequencies = 0.008566 (BI). The best tree obtained from the ML analysis with bootstrap values for BP, BS and BPP is shown in Fig. 2. The phylogeny results in similar topology to the phylogeny based on the ITS + partial nLSU sequences, which supports four new species separated from *T.fibulifera* s.s. and *T.* “*neofibulifera*”.

### Taxonomy

#### Tremella fibulifera

Taxon classificationFungiTremellalesTremellaceae

Möller, Botanische Mittheilungen aus den Tropen 8: 170 (1895)

084FF974-8F91-5300-BE30-11470F2DC526

Figs 3A , 4

##### Basidioma.

Sessile, when fresh gelatinous, pale whitish, lobed to irregularly cerebriform, becoming pale yellowish when dry, 0.5–2.5 cm in diameter, broadly attached to substratum.

##### Internal features.

Hyphae hyaline, smooth, thin- to thick-walled, 2.0–5.0 µm in diameter, branched, interwoven, with abundant clamp connections and medallion clamp connections (clamp complexes), thick-walled hyphae usually present near to base of basidioma; hyphidia hyaline, smooth, thin-walled, branched; swollen cells, vesicles and haustoria absent; mature basidia thin-walled, globose to subglobose, with a basal clamp connection, 13.0–18.0(–22.0) × 9.0–16.0 μm, L = 15.7 µm, W = 14.8 µm, Q = 1.06 (n = 30/1), sometimes their width greater than length, usually longitudinally septate, rarely obliquely septate, 2–4-celled, with obvious oil drops; sterigmata up to 100 μm long, 1.5–2.0 μm in diameter, slightly protuberant at apex; probasidia thin-walled, subglobose to ellipsoid, mostly proliferating directly from basidial clamps; basidiospores hyaline, thin-walled, mostly ellipsoid to slightly ovoid, apiculate, with oil drops, 7.0–10.0 × 6.0–7.0 μm, L = 8.4 µm, W = 6.5 µm, Q = 1.29–1.40 (n = 60/2), germinating by germ tubes or secondary spores; conidia occasionally present in cluster, originating from conidiophores, hyaline, thin-walled, ellipsoid to subglobose, 2.0–3.0 × 1.0–2.5 μm.

##### Specimens examined.

Brazil Rondônia, Municipality of Jaru, in mixed forest near the airport, 9°40'S, 61°50'W, on wood, associated with old pyrenomycete stromata and litter, 10 October 1986, M. Capelari & R. Maziero 944 (SP211759, duplicate BJFC028110); Pernambuco, Recife, Jardim Botânico do Recife, on angiosperm wood, 16 May 2017, R. L. M. Alvarenga 471 (URM).

##### Notes.

*Tremellafibulifera* was probably a species complex including *T.olens* originally from Australia and *T.neofibulifera* originally from Japan because they shared cerebriform whitish basidioma and abundant clamp complexes (Möller 1895; Bandoni and Oberwinkler 1983; Malysheva et al. 2015). Two specimens (SP211759, Alvarenga 471) from Brazil bearing the common feature of the complex formed a distinct lineage in our phylogenies (Figs 1, 2). Morphologically, the two specimens agree well with *T.fibulifera* except for the presence of conidia (Table 3). However, conidia are unstable in *T.fibulifera*. Möller (1895) described the anamorph of *T.fibulifera*, but the conidia were not observed when Bandoni and Oberwinkler (1983) re-described *T.fibulifera* based on the type designated by Möller (1895). Furthermore, *T.fibulifera* was originally described from Blumenau, Brazil, which is very close to the location of SP211759, Rondônia, Brazil. Therefore, we treat Alvarenga 471 and SP211759 as the representatives of *T.fibulifera* s.s. In addition, *T.fibulifera* s.s. are different from *T.subfibulifera* and *T.australe* by 8.51%, 9.87% sequence differences in the ITS sequences and 2.10%, 1.57% in the partial nLSU sequences respectively.

**Figure 3.**
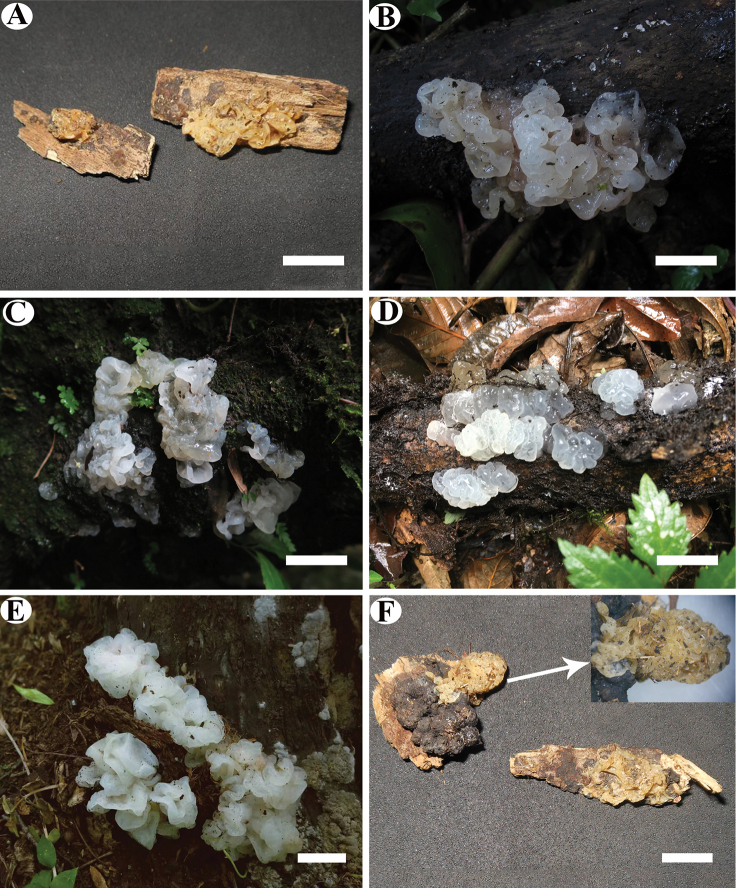
Basidioma **A***Tremellafibulifera* (Alvarenga 471) **B***T.australe* (Wu 154) **C***T.guangxiensis* (Wu 3) **D***T.latispora* (Dai 17568) **E***T.* “*neofibulifera*” (Wu 244) **F***T.subfibulifera* (Alvarenga 334). Scale bars: 1 cm (**A–F**).

**Figure 4.**
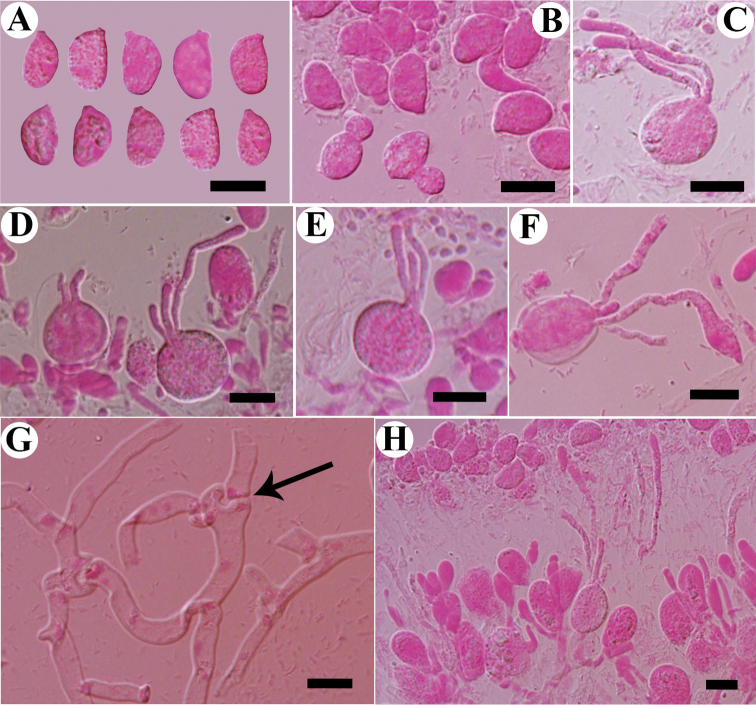
Microscopic structures of *Tremellafibulifera* s.s. (SP 211759) **A** basidiospores **B** germination tubes of basidiospores and secondary spores **C–F** basidia at different stages **G** hyphae with clamp connections and clamp complexes; **H** a section of hymenium. Scale bars: 10 μm (**A–H**).

**Table 3.** A morphological comparison of taxa in the *Tremellafibulifera* complex.

Taxa	Basidia (µm)	Basidiospores (µm)	Conidia (µm)	Hyphidia	Distribution	Reference
* T. fibulifera *	12.0–16.0	7.0–10.0	3.5	Unknown	Brazil	Möller 1895
* T. fibulifera *	15.0–18.0 × 9.0–13.0	8.0–9.0 × 5.0–8.0	Not observed	Unknown	Brazil	Bandoni and Oberwinkler 1983
*T.fibulifera* s.s.	13.0–18.0 × 9.0–16.0	7.0–10.0 × 6.0–7.0	2.0–3.0 × 1.0–2.5	Branched	Brazil	Present study
* T. australe *	14.0–19.0 × 13.0–17.0	8.0–10.0 × 6–8.0	Absent	Present	China	Present study
* T. cheejenii *	12.0–17.0 × 13.0–18.0	5.0–10.0 × 4.5–8.0	2.2–4.0 × 1.8–3.0	Branched	China	Zhao et al. 2019
* T. guangxiensis *	14.0–17.0 × 14.0–16.0	8.0–9.5 × 6.0–7.5	2.0–3.2 × 1.8–3.0	Branched	China	Present study
* T. latispora *	17.2–24.0 × 17.0–23.0	10.1–11.8 × 9.9–11.4	2.8–3.6 × 1.8–3.0	Present	China	Present study
* T. lloydiae-candidae *	14.0–20.0 × 13.0–16.0	7.5–10	Absent	Unknown	Japan, Russia	Malysheva et al. 2015
* T. olens *	Unknown	12.7–14.5	Absent	Unknown	Australia	Hooker 1860
* T. neofibulifera *	13.2–15.5 × 9–10	5.5–8.5 × 4.5–5.5	Absent	Unknown	Japan	Kobayasi 1939
*T.* “*neofibulifera*”	14.0–16.0 × 13.0–17.0	8.0–10.0 × 6.0–8.0	Absent	Parallel	China, Russia	Present study
* T. subfibulifera *	14.4–20.3 × 12.8–16.3	5.4–9.8 × 4.2–6.0	2.0–3.0 × 0.5–1.0	Absent	Brazil	Present study

#### Tremella australe

Taxon classificationFungiTremellalesTremellaceae

F. Wu, L.F. Fan & Y.C. Dai sp. nov.

E330B6AE-51B3-590D-87BE-B946D880128D

839825

Figs 3B , 5

##### Holotype.

China Yunnan, Ruili, on fallen angiosperm branch, 23 April 2018, F. Wu 154 (BJFC028064).

##### Etymology.

Refers to the distribution of this species in South Asia.

##### Basidioma.

Sessile, when fresh soft gelatinous, creamy-white to beige, translucent, cerebriform, with thick and undulate lobes, up to 4.0 cm long, 2.0 cm broad and 2.0 cm high from base, distinctly shrinking into a film and becoming pale yellow when dry, broadly attached to substratum.

##### Internal features.

Hyphae hyaline, smooth, thin- to slightly thick-walled, 1.5–6.0 µm in diameter, branched, interwoven, with abundant clamp connections, clamp complexes and anastomoses, slightly thick-walled hyphae usually present near to base of basidioma and sometimes swollen up to 8.5 μm; hyphidia hyaline, smooth, thin-walled, usually derived from the same hyphae with basidia; swollen cells, vesicles and haustoria absent; mature basidia thin-walled, globose to subglobose, with a basal clamp connection, 14.0–19.0 × 13.0–17.0(–18.0) μm, L = 16.3 µm, W = 15.8 µm, Q = 1.03 (n = 30/1), sometimes their width greater than length, usually longitudinally septate, 2–4-celled, with obvious oil drops; sterigmata up to 20 μm long, 1.0–2.5 in diameter, slightly protuberant at apex; probasidia thin-walled, globose to subglobose, mostly proliferating directly from basidial clamps; basidiospores hyaline, thin-walled, broadly ellipsoid to ellipsoid, apiculate, with oil drops, 8.0–10.0 × 6.0–8.0 μm, L = 8.6 µm, W = 7.3 µm, Q = 1.18–1.28 (n = 60/2), germinating by germ tubes or secondary spores; conidia absent.

**Figure 5.**
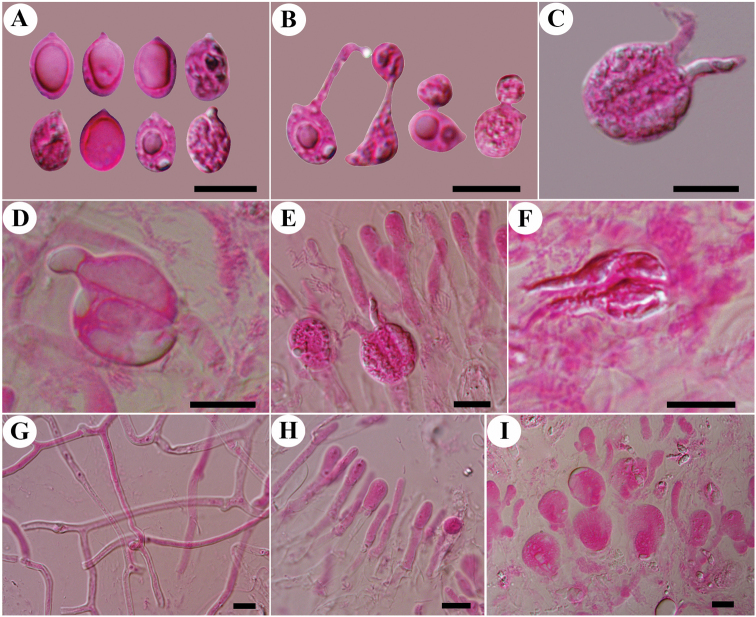
Microscopic structures of *Tremellaaustrale* (Wu 154) **A** basidiospores **B** germination tubes of basidiospores and secondary spores **C–F** basidia at different stages **G** hyphae with clamp connections and clamp complexes **H** hyphidia **I** a section of hymenium. Scale bars: 10 μm (**A–I**).

##### Additional specimen examined.

(***paratype***) China Taiwan, Yilan, Linmei Road, on fallen angiosperm branch, 20 June 2009, Y.C. Dai 11539 (BJFC007408).

##### Notes.

*Tremellaaustrale* formed an independent lineage with high support in our phylogenies (Figs 1, 2). The species is easily confused with *T.guangxiensis* by sharing whitish, translucent cerebriform basidioma and similar basidia and basidiospores, but *T.guangxiensis* has branched hyphidia and umbelliform conidiophores. Besides, *T.australe* are different from *T.subfibulifera*, *T.guangxiensis* and *T.* “*neofibulifera*” by 7.82%, 5.94% and 6.82% sequence differences in the ITS sequences and 2.13%, 3.43% and 1.25% in the partial nLSU sequences respectively.

#### Tremella guangxiensis

Taxon classificationFungiTremellalesTremellaceae

F. Wu, L.F. Fan & Y. C. Dai sp. nov.

BD36A161-CA43-56F5-A585-02532CFB2954

839827

Figs 3C , 6

##### Holotype.

China. Guangxi, Jinxiu, Dayao Mountain, on angiosperm tree, 15 July 2017, F. Wu 3 (BJFC026009).

##### Etymology.

Refers to the distribution of the species in Guangxi, China.

##### Basidioma.

Sessile, when fresh soft gelatinous, milky to creamy-white, translucent, pustulate to irregularly cerebriform, with thick and undulate lobes, up to 4.0 cm long, 4.0 cm broad and 1.5 cm high from base, distinctly shrinking into a film and becoming lightly yellowish when dry, broadly attached to substratum.

##### Internal features.

Hyphae hyaline, smooth, thin- to slightly thick-walled, 2.0–6.0 µm in diameter, branched, interwoven, with abundant clamp connections, clamp complexes and anastomoses, slightly thick-walled hyphae usually present near to base of basidioma and sometimes swollen up to 9.0 μm; hyphidia hyaline, smooth, thin-walled, branched; swollen cells present, hyaline, smooth and various in the shape, sometimes slightly concave; vesicles and haustoria absent; mature basidia thin-walled, globose to subglobose, with a basal clamp connection, 14.0–17.0 × (13.6–)14.0–16.0(–17.0) μm, L = 15.9 µm, W = 14.8 µm, Q = 1.07 (n = 30/1), sometimes their width greater than length, usually longitudinally septate, rarely obliquely septate, 2–4-celled, with obvious oil drops; sterigmata up to 60 μm long, 1.5–2.0 in diameter, slightly protuberant at apex; probasidia thin-walled, clavate to ellipsoid, proliferating from terminal hyphae; basidiospores hyaline, thin-walled, broadly ellipsoid to slightly ovoid, apiculate, with oil drops, (7.5–)8.0–9.5 × 6.0–7.5(–8.0) μm, L = 8.7 µm, W = 6.8 µm, Q = 1.28 (n = 30/1), germinating by germ tubes or secondary spores; conidia massively present, originating from umbelliform conidiophores, hyaline, thin-walled, ovoid to broadly ellipsoid or fusiform to cylindrical, 2.0–3.2 × 1.8–3.0 μm.

**Figure 6.**
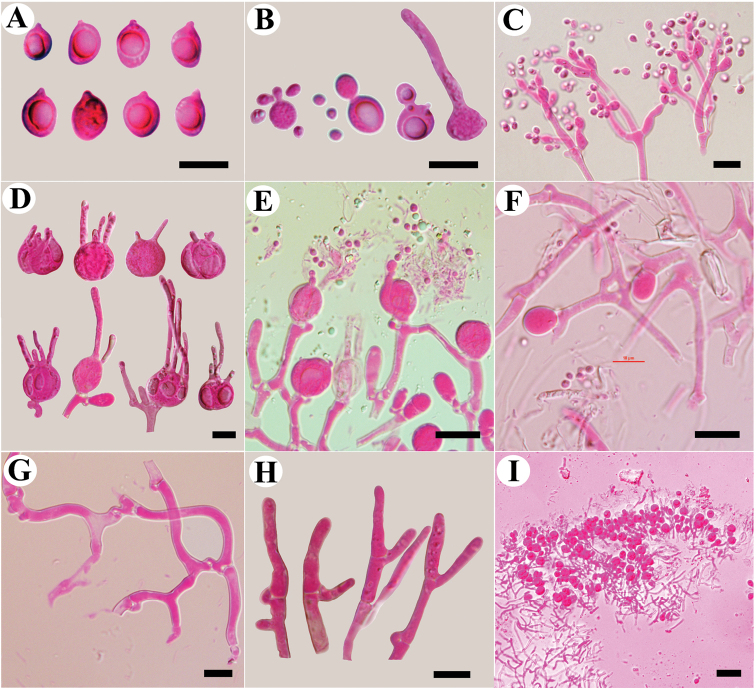
Microscopic structures of *Tremellaguangxiensis* (Wu 3) **A** basidiospores **B** germination tubes of basidiospores and secondary spores **C** conidia and conidiophores **D** basidia at different stages **E, F** probasidia **G** hyphae with clamp connections and clamp complexes **H** hyphidia **I** a section of hymenium. Scale bars: 10 μm (**A–H**); 20 μm (**I**).

##### Notes.

*Tremellaguangxiensis* is closely related *T.* “*neofibulifera*” in our phylogenies (Figs 1, 2). The most distinctive characteristic of the species is branched hyphidia and umbelliform conidiophores, but *T.* “*neofibulifera*” has parallel hyphidia and lacks of conidia. In addition, *T.guangxiensis* are different from *T.australe* and *T.* “*neofibulifera*” by 6.35% and 5.09% sequence differences in the ITS sequences and 3.39% and 1.97% in the partial nLSU sequences respectively.

#### Tremella latispora

Taxon classificationFungiTremellalesTremellaceae

F. Wu, L.F. Fan & Y. C. Dai sp. nov.

AD8B8676-A659-5FB1-946D-E77E706D13AC

839828

Figs 3E , 7

##### Holotype.

China. Yunnan, Xinping, Shimenxia Park, on stump of *Lithocarpus*, 16 June 2017, Y.C. Dai 17574 (BJFC025106).

##### Etymology.

Refers to the species having wide basidiospores.

##### Basidioma.

Sessile, when fresh soft gelatinous, creamy-white to lvory, translucent, pustulate to irregularly cerebriform, with thick and undulate lobes, up to 4.0 cm long, 2.0 cm broad and 1.0 cm high from base, distinctly shrinking into a film and becoming whitish to pale yellow when dry, broadly attached to substratum.

##### Internal features.

Hyphae hyaline, smooth, thin- to thick-walled, 1.5–6.0 µm in diameter, branched, interwoven, with abundant clamp connections, clamp complexes and anastomoses, thick-walled hyphae usually present near to base of basidioma and sometimes swollen up to 7.5 μm; hyphidia hyaline, smooth, thin-walled, usually derived from the same hyphae with basidia; swollen cells, vesicles and haustoria absent; mature basidia thin-walled, globose to subglobose, with a basal clamp connection, 17.2–24.0(–27.0) × 17.0–23.0(–24.3) μm, L = 19.5 µm, W = 20.8 µm, Q = 0.94 (n = 30/1), commonly their width greater than length, usually longitudinally septate, occasionally obliquely septate, 2–4-celled, with obvious oil drops; sterigmata up to 60 μm long, 1.5–2.0 in diameter, slightly protuberant at apex; probasidia thin-walled, ellipsoid to subglobose, proliferating from terminal hyphae; basidiospores hyaline, thin-walled, globose to subglobose, apiculate, with oil drops, (9.0–)10.1–11.8(–12.0) × (9.6–)9.9–11.4(–11.7) μm, L = 11.0 µm, W = 10.7 µm, Q = 1.03 (n = 30/1), germination by germ tubes or secondary spores; conidia massively present, originating from umbelliform conidiophores, hyaline, thin-walled, ovoid to oblong or globose to subglobose, 2.8–3.6 × 1.8–3.0 μm.

**Figure 7.**
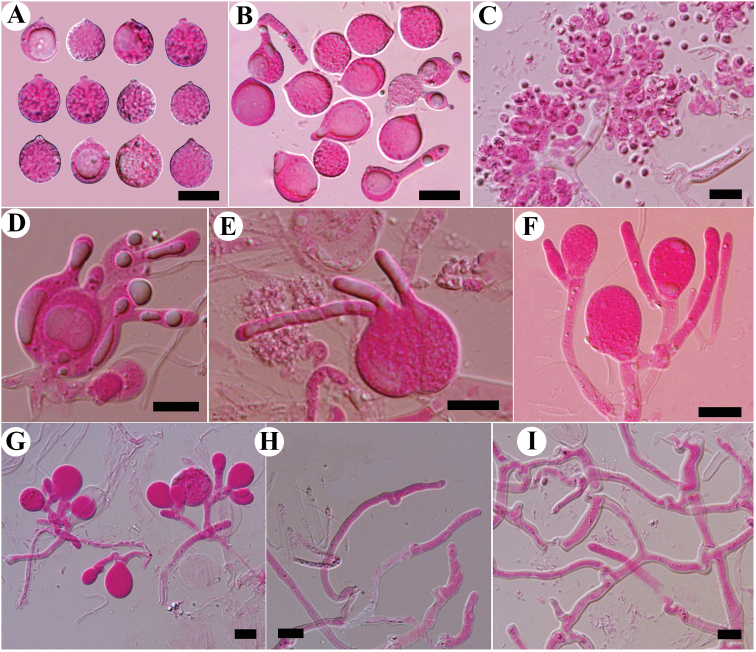
Microscopic structures of *Tremellalatispora* (Dai 17568) **A** basidiospores **B** germination tubes of basidiospores and secondary spores **C** conidia and conidiophores **D, E** basidia **F, G** probasidia **H, I** hyphae with clamp connections and clamp complexes. Scale bars: 10 μm (**A–I**).

##### Additional specimen examined.

(***paratype***) China Yunnan, Xinping, Shimenxia Park, on stump of *Lithocarpus*, 16 June 2017, Y.C. Dai 17568 (BJFC025100).

##### Notes.

Phylogenetically, *Tremellalatispora* formed a distinct lineage closely related to *T.cheejenii* (Figs 1, 2). Morphologically, the species has significantly larger basidia and basidiospores than *T.cheejenii* or other similar species (Table 3), and it has globose to subglobose basidiospores rather than more or less ellipsoid basidiospores in other species. And *T.latispora* are different from *T.cheejenii* and *T.fibulifera* s.s. by 4.63% and 5.09% sequence differences in the ITS sequences and 3.39% and 2.95% in the partial nLSU sequences respectively.

#### T. neofibulifera

Taxon classificationFungiTremellalesTremellaceae

Kobayasi, Scientific Report, Tokyo Bunrika Daigaku, Section 4: 15 (1939)

058D9F07-1D89-5218-994C-048A93A16083

Figs 3D , 8

##### Basidioma.

Sessile, when fresh soft gelatinous, creamy-white to pale yellowish, irregularly cerebriform or slightly foliose, with undulate lobes, up to 4.5 cm long, 2.0 cm broad and 2.5 cm high from base, becoming firmly gelatinous and invisible yellowish when dry, broadly attached to substratum.

##### Internal features.

Hyphae hyaline, smooth thin- to slightly thick-walled, 2.0–6.0 µm in diameter, branched, interwoven, with abundant clamp connections, clamp complexes and anastomoses, slightly thick-walled hyphae usually present near to base of basidioma, sometimes swollen up to 8.5 μm; hyphidia hyaline, smooth, thin-walled, arranged in cluster, usually parallel; vesicles infrequent, thick-walled; swollen cells and haustoria absent; mature basidia thin-walled, ovoid to subglobose, with a basal clamp connection, 14.0–16.0 × 13.0–17.0 μm, L = 14.9 µm, W = 14.8 µm, Q = 1.01 (n = 30/1), sometimes their width greater than length, usually longitudinally septate, rarely obliquely septate, 2–4-celled, with obvious oil drops; sterigmata up to 70 μm long, 1.5–2.0 in diameter, slightly protuberant at apex; probasidia thin-walled, ellipsoid to subglobose, usually proliferating from terminal hyphae; basidiospores hyaline, thin-walled, ellipsoid to broadly ellipsoid, apiculate, with oil drops, 8.0–10.0 × 6.0–8.0 μm, L = 8.9 µm, W = 6.5 µm, Q = 1.37 (n = 30/1), germination by germ tubes or secondary spores; conidia absent.

**Figure 8.**
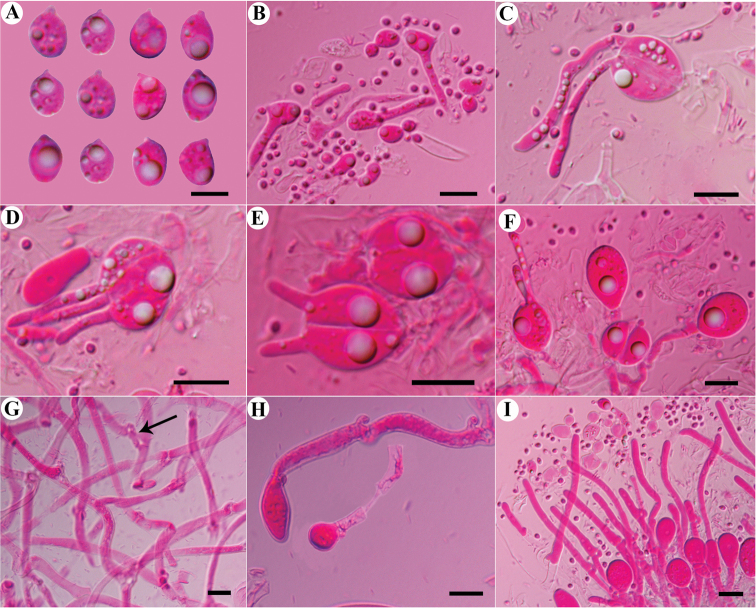
Microscopic structures of *Tremella “neofibulifera*” (Wu 248) **A** basidiospores **B** germination tubes of basidiospores and secondary spores **C–E** basidia at different stages **F** probasidia **G** hyphae with clamp connections and clamp complexes **H** vesicles **I** parallel hyphidia in hymenium. Scale bars: 10 μm (**A–I**).

##### Specimens examined.

China Jilin, Helong, Quanshuidong Forest Farm, on stump of *Quercus*, 15 July 2017, F. Wu 243 (BJFC031046); F. Wu 244 (BJFC031047); F. Wu 248 (BJFC031051).

##### Notes.

Three specimens listed above from Northeast China together with LE303445 from Far East of Russia formed a distinct lineage closely related to *T.guangxiensis* in our phylogenies (Figs 1, 2). *T.neofibulifera* was originally described from Japan (Kobayasi 1939), and our studied East Asian samples have similar morphology to *T.neofibulifera* except bigger basidiospores (Table 3). We fail to loan the type of *T.neofibulifera*, and for the time being we treat our studied East Asia samples as *T.* “*neofibulifera*”. The current *T.* “*neofibulifera*” differs from other similar species of the *Tremellafibulifera* complex by the parallel hyphidia and the presence of vesicles. In addition, *T.* “*neofibulifera*” are different from *T.guangxiensis*, *T.australe*, *T.subfibulifera* and *T.fibulifera* s.s. by 3.15%, 5.25%, 7.14%, and 8.19% sequence differences in the ITS sequences and 2.04%, 1.32%, 3.18%, and 2.41% in the partial nLSU sequences respectively.

#### Tremella subfibulifera

Taxon classificationFungiTremellalesTremellaceae

Alvarenga, F. Wu, L.F. Fan & Y.C. Dai sp. nov.

6385C3CD-A50F-5D7A-9406-A5A84F19EF9B

839829

Figs 3F , 9

##### Holotype.

Brazil. Pernambuco, Recife, Jardim Botânico do Recife, on angiosperm wood, 17 June 2016, R. L. M. Alvarenga 334 (URM).

##### Etymology.

Refers to the species being similar to *Tremellafibulifera*.

##### Basidioma.

Sessile, when fresh gelatinous, pale white, foliose to irregularly cerebriform, with undulate lobes, up to 3.0 cm long, 2.0 cm broad and 1.0 cm high from base, becoming firmly gelatinous and pale yellowish when dry, broadly attached to substratum.

##### Internal features.

Hyphae hyaline, smooth, slightly thick-walled, 2.0–4.0 μm in diameter, branched, interwoven, with abundant clamp connections, clamp complexes and anastomoses; hyphidia, swollen cells, vesicles and haustoria absent; mature basidia thin-walled, subglobose to broadly ellipsoid, with a basal clamp connection, (14.0–)14.4–20.3(–21.0) × (9.0–)12.8–16.3(–17.8) μm, L = 17.63 µm, W = 15.05 µm, Q = 1.17 (n = 30/1), sometimes their width greater than length, usually longitudinally or obliquely septate, 2–4-celled, with obvious oil drops; mature sterigmata often collapsed, juvenile sterigmata up to 15.0 µm long, 2.0–4.0 µm in diameter, slightly protuberant at apex; probasidia thin-walled, clavate to ellipsoid, guttulate, proliferating from terminal hyphae; basidiospores hyaline, thin-walled, ellipsoid apiculate, with oil drops, (5.0–)5.4–9.8(–10.0) × (4.0–)4.2–6.0(–6.4) μm, L = 8.0 µm, W = 5.3 µm, Q = 1.50 (n = 30/1); conidia massively present, originating from umbelliform conidiophores, hyaline, thin-walled, variously shaped, ellipsoid, fusiform to cylindrical, 2.0–3.0 × 0.5–1.0 μm.

**Figure 9.**
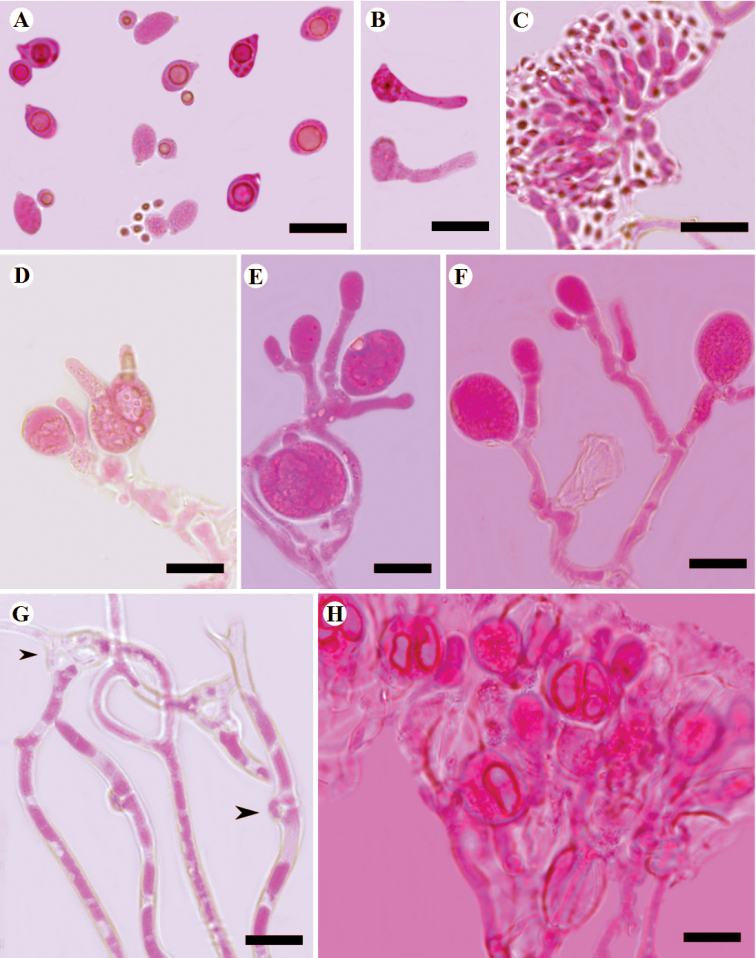
Microscopic structures of *Tremellasubfibulifera* (Alvarenga 334) **A** basidiospores and secondary spores **B** germination tubes of basidiospores **C** conidia and conidiophores **D–F** basidia and probasidia **G** hyphae with clamp connections and clamp complexes **H** a section of hymenium. Scale bars: 10 μm (**A–H**).

##### Notes.

*Tremellasubfibulifera* nested in the clade of the *T.fibulifera* complex, and formed an independent lineage. It resembles *T.fibulifera* s.s., but *T.fibulifera* s.s. has larger basidiospores (7.0–10.0 × 6.0–7.0 μm vs. 5.4–9.8 × 4.2–6.0 μm) and the presence of branched hyphidia (Table 3). In addition, *T.subfibulifera* are different from *T.australe* and *T.fibulifera* s.s. by 6.19% and 7.85% sequence differences in the ITS sequences and 2.23% and 2.10% in the partial nLSU sequences respectively.

## Discussion

*Tremellafibulifera* was originally described from Blumenau of Brazil (Möller 1895); later two similar species, *T.olens* and *T.neofibulifera*, were respectively described from Tasmania of Australia and Simotuke of Japan (Hooker 1860; Kobayasi 1939). The Russian Far East specimen LE303445 was identified as *T.fibulifera* by Malysheva et al. (2015). Our results demonstrated the Northeastern Chinese specimens and Russian Far East specimen formed an independent lineage, and this lineage is distantly related to the lineage formed by two Brazilian specimens, SP 211759 and Alvarenga 471 (Figs 1, 2). The location of SP 211759 is near to the type locality of *T.fibulifera*. So, we treat SP 211759 and Alvarenga 471 as representatives of *T.fibulifera* s.s. Molecular data are not available from type or type locality specimens of *T.neofibulifera*. Neither is its type re-examined, but the Northeastern Chinese specimens have more or less similar morphology as the description of *T.neofibulifera*, so we temporarily treat Northeast Chinese specimens and Russian Far East specimen as *T.* “*neofibulifera*”.

The Southern Chinese specimen GX20172028 was also identified as *Tremellafibulifera* by Zhao et al. (2019), but it clustered with another Southern Chinese specimen Wu 3 into a distinct lineage which is closely related to *T.* “*neofibulifera*” (Figs 1, 2). *T.guangxiensis* is different from *T.* “*neofibulifera*” by 5.09% sequence differences in the ITS sequences and 1.97% in the partial nLSU sequences respectively. In addition, the Southern Chinese specimens have translucent basidioma, branched hyphidia and umbelliform conidiophores, and they are readily distinguished from *T.* “*neofibulifera*”. So, these two specimens are identified as a new species *T.guangxiensis*.

Seven species, *Tremellafibulifera*, *T.olens*, *T.* “*neofibulifera*”, *T.guangxiensis*, *T.australe*, *T.latispora* and *T.subfibulifera* have cerebriform whitish basidioma and abundant clamp complexes, and they nested in the same clade. So, we treat these seven species as members of the *T.fibulifera* complex.

*Tremellalloydiae-candidae* Wojewoda and *T.cheejenii* Xin Zhan Liu & F.Y. Bai also have whitish basidioma and similar micro-morphology with *T.fibulifera*, but clamp complexes were not observed (Malysheva et al. 2015; Zhao et a. 2019), and we did not examine their types. Because these two species are nested in the same clade as other species of the *T.fibulifera* complex with robust support in our phylogenies (Figs 1, 2), we treat them as members of the *T.fibulifera* complex, too.

Currently, more than 30 morphological characteristics are applied for identification species of *Tremella* s.s. (Chen 1998; Zhao et al. 2019), and some features including basidioma color and basidia shape are variable at different stages. The shape and size of basidiospores are relatively stable characteristics for each species, but they are very similar among some species in the *T.fibulifera* complex; that is why several taxa were previously treated as *T.fibulifera* s.l. (Malysheva et al. 2015; Zhao et al. 2019). Consequently, combined morphology and molecular evidence are essential to distinguish species within the complex, and ITS + partial nLSU dataset are selected for species delimitation.

### Key to the whitish species in *Tremella* s. s.

**Table:** 

1	Basidiospores > 10 μm long	**2**
–	Basidiospores < 10 μm long	**5**
2	Basidioma resupinate	*** T. resupinata ***
–	Basidioma pustulate to irregularly cerebriform or foliose	**3**
3	Basidiospores > 17 μm long	*** T. cerebriformis ***
–	Basidiospores < 17 μm long	**4**
4	Basidiospores > 12 μm long	*** T. olens ***
–	Basidiospores < 12 μm long	*** T. latispora ***
5	Basidia with stalks	**6**
–	Basidia without stalks	**8**
6	Basidia < 13 μm wide	*** T. yakohamensis ***
–	Basidia > 13 μm wide	**7**
7	Basidiospores mostly broader than long	*** T. globispora ***
–	Basidiospores mostly longer than broad	*** T. cheejenii ***
8	Basidia with sterigmata shorter than 35 μm	**9**
–	Basidia with sterigmata longer than 35 μm	**11**
9	Basidiospores < 6 μm wide	*** T. subfibulifera ***
–	Basidiospores > 6 μm wide	**10**
10	Hyphae with clamp complexes and anastomoses	*** T. australe ***
–	Hyphae without clamp complexes and anastomoses	*** T. lloydiae-candidae ***
11	Basidioma filamentous lobes, conjunctive as a ball	*** T. hainanensis ***
–	Basidioma pustulate to irregularly cerebriform or foliose	**12**
12	Basidiospores < 6 μm wide	*** T. fuciformis ***
–	Basidiospores > 6 μm wide	13
13	Hyphidia parallel; conidia absent	***T.* “*neofibulifera* ”**
–	Hyphidia branched; conidia present	**14**
14	Basidioma pustulate to irregularly cerebriform; basidia with sterigmata up to 60 μm; conidia originating from umbelliform conidiophores	*** T. guangxiensis ***
–	Basidioma lobed to irregularly cerebriform; basidia with sterigmata up to 100 μm; conidia not originating from umbelliform conidiophores	***T.fibulifera* s.s.**

## Supplementary Material

XML Treatment for Tremella fibulifera

XML Treatment for Tremella australe

XML Treatment for Tremella guangxiensis

XML Treatment for Tremella latispora

XML Treatment for T. neofibulifera

XML Treatment for Tremella subfibulifera
